# Cost-effectiveness analysis of isolation strategies for asymptomatic and mild symptom COVID-19 patients

**DOI:** 10.1186/s12962-023-00497-x

**Published:** 2023-11-09

**Authors:** Unyaporn Suthutvoravut, Patratorn Kunakorntham, Anchisatha Semayai, Amarit Tansawet, Oraluck Pattanaprateep, Pongsathorn Piebpien, Pawin Numthavaj, Ammarin Thakkinstian, Pongsakorn Atiksawedparit

**Affiliations:** 1 Department of Family medicine, Faculty of Medicine Ramathibodi Hospital, Mahidol University, Bangkok, Thailand; 2 Department of Information Technology, Faculty of Medicine Ramathibodi Hospital, Mahidol University, 270 Rama VI Rd, Ratchathewi, Bangkok, 10400 Thailand; 3 Department of Finance, Faculty of Medicine Ramathibodi Hospital, Mahidol University, Bangkok, Thailand; 4 Department of Clinical Epidemiology and Biostatistics, Faculty of Medicine Ramathibodi Hospital, Mahidol University, Bangkok, Thailand; 5 Department of Surgery, Faculty of Medicine Vajira Hospital, Navamindradhiraj University, Bangkok, Thailand; 6 Faculty of Medicine Ramathibodi Hospital, Chakri Naruebodindra Medical Institute, Mahidol University, Samut Prakan, Thailand

**Keywords:** Cost-effectiveness analysis, Isolation strategies, Asymptomatic, COVID-19

## Abstract

**Background:**

Management of COVID-19 patients with mild and moderate symptoms could be isolated at home isolation (HI), community isolation (CI) or hospitel. However, it was still unclear which strategy was more cost-effective. Therefore, this study was conducted to evaluate this.

**Methods:**

This study used data from patients who initially stayed at HI, CI, and hospitel under supervision of Ramathibodi Hospital between April and October 2021. Outcomes of interest were hospitalisation and mortality. An incremental cost-effectiveness ratios (ICER) was calculated based on hospital perspective using home isolation as the reference.

**Results:**

From 7,077 patients, 4,349 2,356, and 372 were admitted at hospitel, HI, and CI, respectively. Most patients were females (57.04%) and the mean age was 40.42 (SD = 16.15). Average durations of stay were 4.47, 3.35, and 3.91 days for HI, CI, and hospitel, respectively. The average cost per day for staying in these corresponding places were 24.22, 63.69, and 65.23 US$. For hospitalisation, the ICER for hospitel was at 41.93 US$ to avoid one hospitalisation in 1,000 patients when compared to HI, while CI had more cost, but less cases avoided. The ICER for hospitel and CI were at 46.21 and 866.17 US$ to avoid one death in 1,000 patients.

**Conclusions:**

HI may be cost-effective isolated strategy for preventing hospitalisation and death in developing countries with limited resources.

**Supplementary Information:**

The online version contains supplementary material available at 10.1186/s12962-023-00497-x.

## Introduction

COVID-19 outbreak was first identified in Wuhan, Hubei province, China in 2019 and later spread all over the world [1, 2]. The clinical symptoms of COVID-19 ranges from asymptomatic to symptomatic including fever, cough, fatigue, sore throat, dyspnea, diarrhea, headache and loss of taste and smell [3, 4]. The risk of acute respiratory distress syndrome was about 9.4% with mortality rate of 3.2% [5]. The majority of patients are asymptomatic or have mild symptoms [6, 7]. The virus is highly transmitted via droplets and fomites [8–10], thus, patients should be immediately isolated to prevent viral transmission [11].

WHO guidance recommended that the isolation of the patients with mild to moderate symptoms could be done at an appointed COVID-19 health facility, community facility, or by self-isolation at home depending on the care pathway [12]. Isolation hotel, known as hospitel, is also another option for patients who experience homelessness during COVID-19 infection and this strategy could also reduce hospital admission [13].

Thailand was the first country outside China to report the first case of laboratory confirmed COVID-19 on January 13th, 2020, for a Chinese woman from Wuhan [14]. Later, the first local human-to human transmission of COVID-19 in a Thai taxi driver was reported on January 31st, 2020 [15]. The Thai government officially declared COVID-19 as a dangerous communicable disease under the Ministry of Public Health (MOPH)’s communicable disease ACT B.E 2558 (2015). On March 26th, 2020 all areas of the Kingdom of Thailand were declared by the Thai Prime Minister to be pursuant to the Emergency Decree on Public Administration in emergency situations [16]. Measures and regulations of the Thai government included restriction of international travel, screening all ports of entry and quarantine of travellers. Guidelines related to COVID-19 were developed and distributed to medical and non-medical personnel by the MOPH. Multiple strategies were used to manage patients and relieve pressure for hospital capacity including hospitel, home isolation (HI), and community isolation (CI). The hospitel was transformed from vacant hotels during the pandemic by the collaboration of public administrations, private and public hospitals, and hotels [17]. HI, targeted on low-risk patients, has been implemented with special technology for remote tracking and warning system [18]. CI facilities were set up to look after low-risk patients who could not isolate themselves at home [19]. It was operated by collaboration of people who lived in that community and hospital, whilst some facilities were also equipped with mobile X-ray vehicles [20].

Ramathibodi Hospital, is a tertiary care-university hospital under Mahidol University located in Bangkok. During the COVID-19 outbreak, multidisciplinary working groups and strategies had been set up to cope with the situation including care pathway, preparation of care facilities, medical equipment, personnel/public education, and COVID-19 call center. In addition, the HI, CI, and hospitel were care facilities that could operate with joint referral systems between out-hospital facilities and hospital. Each setting had different additional costs, i.e., costs of renting hotels included dietary for hospitel, cost of building renovation (disposable items) for CI, and dietary costs for both HI and CI.

Given the constraints of health-care resources, the cost-effectiveness of these strategies in management of the outbreak was very important. A systematic review of cost-effectiveness of COVID-19 policy measures was conducted by including only a few studies which investigated the treatment strategies [21]. Sheinson et al. showed that treatment for COVID-19 hospitalised patients were cost-effective under a health payer, a societal, and a fee-for-service (FFS) payment model perspective when compared to supportive care [22]. A study from South Africa found that the incremental cost-effectiveness ratio of general ward versus general ward plus intensive care was ZAR 73,091 per disability adjusted life year, which averted disabilities was below threshold, showing non-cost-effectiveness [23]. A cost-effectiveness analysis of expanding ICU bed capacity in Germany showed cost-effective if bed utilisation was low [24]. For isolation strategy, there was only one study conducted in Australia which found that HI was cost-effective relative to hospitel quarantine [25]. Furthermore, there was no report of the result of isolation models among low to middle income countries, where health care systems (e.g., health insurance system, healthcare accessibility, or standard of care), economic systems (e.g., incomes, household expenditures, or budget allocation) and societal structures (e.g., level of education, beliefs, or local governance) are different relative to high income countries. Moreover, these might affect to the expense of health care and patient important outcomes. In addition, isolation strategy such as CI has neither been explored for cost-effectiveness nor reviewed, which might be important information for hospital to make decision when facing to new emerging disease. Thus, this cost-effectiveness study was conducted to compare HI, CI, and hospitel isolation strategies with hospital perspective.

## Materials and methods

This was a cross-sectional study focused on cost-effectiveness analysis of HI, CI, and hospitel isolation strategies evaluated based on a causal conceptual frame-work in Supplement Fig. 1. To control the initial disease progression and severity symptoms of patients, only COVID-19 patients with asymptomatic or mild symptoms were included. Patients were enrolled with the following criteria: aged 15 years or older, had been infected with COVID-19 with asymptomatic or mild symptoms, and initially underwent any of the isolation strategies of interest (i.e., HI, CI, and hospitel) provided by Ramathibodi Hospital between 1st April and 31st October 2021. Patients who had been firstly admitted as inpatients at Ramathibodi Hospital were excluded. This study was approved by the Ethic committee, Faculty of Medicine Ramathibodi Hospital, Mahidol University board (COA MURA2021/866).

Once COVID-19 was confirmed, patients with mild symptoms or asymptomatic were isolated at home, or renovated public shelters (e.g., stadium, local government meeting hall or school) and hotels for HI, CI and hospitel isolation, respectively. For HI, healthcare staffs would consider and assess if households had appropriated facilities for isolation to prevent transmission among members of household, i.e., had a separate room with included bathroom, had separated common room and/or kitchen. In addition, patients and family members were emphasised and concerned about the importance of strict standard precautions, basic infectious controls, and not allowed to leave from specified area.

For CI, patients stayed in temporary shelter or restricted areas where were organized and looked after by local governments under supervision of hospital staffs. For hospitel isolation, Ramathibodi Hospital had collaborated with a few hotels nearby and setup isolations under supervisions of nurse and doctor teams. Patients were strictly stayed in their rooms since enter without meeting in persons with other patients. Food, water, and medications were provided and delivered. According to this study’s protocol, standard precaution guide was advised to all patients. Vital signs (i.e., pulse rate, body temperature and pulse oximeter) were daily self-measured and reported to hospital staff by telephone. In addition, patients in HI and hospitel were also daily contacted by hospital staffs via telephone, video call via line application to follow up their sign and symptoms. If worsening of symptom was suspected, patients would be transported to hospital for further evaluation and treatment under the same treatment protocol for HI, CI and hospitel.

**Fig. 1:**
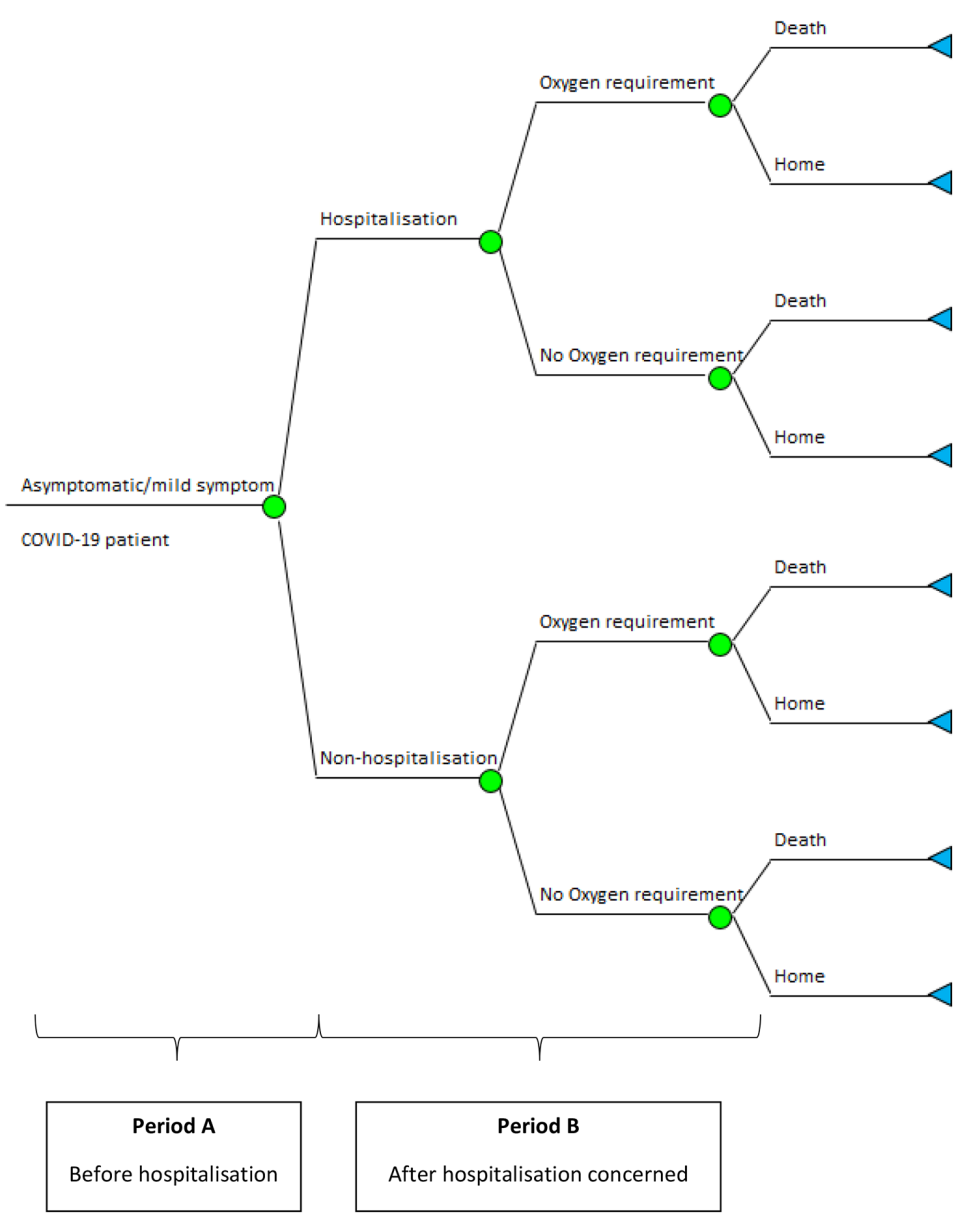
Decision tree model

Baseline characteristics, clinical, and utilisation data of all eligible patients were retrieved from the hospital information system and RamaCare application, which was developed to collect patient daily COVID-19 comorbidities e.g., COPD, diabetes mellitus, etcetera. The intervention of interest was isolation strategy including, HI, CI or hospitel. Two outcomes of interest were hospitalisation and mortality. Cost-effectiveness analysis was performed in hospital perspective for both outcomes based on decision tree model, see Fig. 1. The model started with asymptomatic or mild symptoms COVID-19 patient who were allocated to isolation place, then they were transferred to admit at the hospital if they had more severe symptoms or stayed in the isolation area until discharge (i.e., dead or alive). Patients might need oxygenation either in isolation place or in the hospital.

### Cost measurement

Only direct medical cost was measured by retrieving all facility and medical care utilisations (e.g., room, drug, medical supply, dietary, etcetera) incurred during the treatment period. Three approaches were applied for unit cost calculation, i.e., the University Hospital Network (UHOSNET)’s project, the margins announced from the Comptroller General Department’s guideline, and activity based costing. The UHOSNET covered 89.3% of all costs, which calculated cost per unit based on the summation of variable and fixed costs with complicated allocation criteria by using the total costs of treatment in Ramathibodi Hospital between 1st October 2020 and 30th September 2021. For additional new services created after the UHOSNET’s project those were calculated in accordance with the guideline for drug pricing of the Comptroller General’s Department [25], except the new services related to room or dietary costs which were based on the investment in building, i.e., costs of renting hotels for hospitel, cost of building renovation (disposable items) for CI, no room cost for HI, and unit cost of special room without toilet for hospitalisation. In addition, dietary costs for both HI and CI were at 8.17 US$ per day whereas it was 0.27 US$ per meal for hospitalisation. There was no extra dietary cost for hospitel, since it was already included in rental cost. All costs were converted from Thai Baht to US$ at 36.71 [26] and reported as 2021 value.

### Data analysis – baseline, cost description, cost-effectiveness analysis

Costs (i.e., total cost, cost per admission, and per patient day) were described by isolation strategies. Univariate analysis was performed to compare characteristics of patients (i.e., age, gender, and comorbidities) and costs among the 3 isolation strategies. For modelling, the costs were calculated separately by 2 periods, i.e., (A) before hospitalisation and (B) after hospitalisation, see Fig. 1. For cost outcome, i.e., only cost in period A was considered, while cost of both periods was added for death outcome. Oxygen utilisation was separately calculated per admission since the date of oxygenation could not be identified. In addition, cost of each isolation strategy was categorised by product type and showed by percentage of total treatment cost.

Cost-effectiveness analysis was performed based on the decision tree model by applying cost parameters (cost per patient day x patient day per admission), plus cost of oxygen for each arm, while transitional probabilities were calculated as a ratio of each arm in the decision tree from this study’s raw data see Supplementary Fig. 2. Incremental cost-effectiveness ratios were calculated for surrogate outcome (as US$ per hospitalisation avoidance in 1000) and final outcome (as US$ per death avoidance in 1000), using home isolation as the reference strategy in Microsoft Excel and TreePlan addin. The equation used to calculate the cost and incremental cost-effectiveness ratios (ICER) for each intervention arm were as detail below. The estimated ICERs of three intervention arms were then compared using HI as the reference group.

$$Cost = Cost\, per\, patient\, day \times Patient\, dat\, per\, admission$$

$$Cost = \frac{{Cost}_{exp} - {Cost}_{control}}{{Outcome}_{exp}-{Outcome}_{control}}$$

## Results

A total of 7,077 COVID-19 patients with asymptomatic and mild symptoms were isolated at Ramathibodi facilities between 1st April and 31st October 2021. Of them, 4,349, 2,356 and 372 patients stayed at hospitel, HI, and CI, respectively. Characteristics of these patients are described in Table 1. HI patients seemed to be older and might have more comorbidities (i.e., COPD, cardiovascular disease, diabetes mellitus, ESRD, hypertension, mental illness, and stroke) than the other two groups. Most of patients were enrolled to hospital (61.5%), whereas 33.3% and 5.2% were HI and CI, respectively, see Table 2. The highest hospitalisation rate was found in CI (5.38%), whereas the rates in HI and hospitel were 4.07% and 3.72%, respectively, see Table 3. However, the highest death rate was in HI (8.49/1000), followed by CI and hospitel of 8.06/1000 and 1.61/1000, see Table 4.

**Table 1:** Describe demographic data by Covid-19 isolation strategies

Factor n (%)	Total n = 7,077	Home isolation n = 2,356	Community isolation n = 372	Hospitel n = 4,349	p-value^*^
Age, mean (SD)	40.42 (16.15)	43.89 (17.70)	42.22 (17.94)	38.38 (14.71)	< 0.001
Gender					
Male	3,040 (42.96)	1,011 (42.91)	178 (47.85)	1,,851 (42.56)	0.141
Female	4,037 (57.04)	1,345 (57.09)	194 (52.15)	2498 (57.44)	
Cancer					
Yes	352 (4.97)	146 (6.20)	12 (3.23)	194 (4.46)	0.002
No	6,725 (95.03)	2,210 (93.80)	360 (96.77)	4,155 (95.54)	
COPD					
Yes	217 (3.07)	99 (4.20)	12 (3.23)	106 (2.44)	< 0.001
No	6,860 (96.93)	2,257 (95.80)	360 (96.77)	4,216 (97.56)	
Cardiovascular disease					
Yes	1297 (18.33)	576 (24.45)	69 (18.55)	652 (14.99)	< 0.001
No	5,780 (81.67)	1,780 (75.55)	303 (81.45)	3,697 (85.01)	
Diabetes mellitus					
Yes	353 (4.99)	151 (6.41)	20 (5.38)	182 (4.18)	< 0.001
No	6,724 (95.01)	2,205 (93.59)	352 (94.62)	4,167 (95.82)	
ESRD					
Yes	181 (2.56)	102 (4.33)	4 (1.08)	75 (1.72)	< 0.001
No	6,896 (97.44)	2,254 (95.67)	368 (98.92)	4,274 (98.28)	
HIV					
Yes	22 (0.31)	8 (0.34)	3 (0.81)	11 (0.25)	0.175
No	7,055 (99.69)	2,348 (99.66)	369 (99.19)	4,338 (99.75)	
Hypertension					
Yes	1,126 (15.91)	513 (21.77)	62 (16.67)	551 (12.67)	< 0.001
No	5,951 (84.09)	1,843 (78.23)	310 (83.33)	3,798 (87.33)	
Liver disease					
Yes	148 (2.09)	55 (2.33)	9 (2.42)	84 (1.93)	0.492
No	6,929 (97.91)	2,301 (97.67)	363 (97.58)	4,265 (98.07)	
Mental illness					
Yes	147 (2.08)	77 (3.27)	7 (1.88)	63 ( 1.45)	< 0.001
No	6,930 (97.92)	2,279 (96.73)	365 (98.12)	4,286 (98.55)	
Obesity					
Yes	542 (7.66)	220 (9.34)	30 (8.06)	292 (6.71)	0.001
No	6,535 (92.34)	2,136 (90.66)	342 (91.94)	4,057 (93.29)	
Stroke					
Yes	112 (1.58)	60 (2.55)	6 (1.61)	46 (1.06)	< 0.001
No	6,965 (98.42)	2,296 (97.45)	366 (98.39)	4,303 (98.94)	
Tuberculosis					
Yes	74 (1.05)	18 (0.76)	3 (0.81)	53 (1.22)	0.195
No	7,003 (98.95)	2,338 (99.24)	369 (99.19)	4,296 (98.78)	

*Comparisons were performed by one-way analysis of variance and Chi-square tests for quantitative and categorical data, respectively

**Table 2:** Number of admissions, patient days, total cost, cost per admission, cost per patient day

	Home isolation	Community isolation	Hospitel	Total
Number of admissions	2,356	372	4,349	7,077
Patient days	21,780	3,852	39,218	64,850
Total cost	993,471	293,626	3,216,461	4,503,558
Cost per admission	422	789	740	636
Cost per patient day	46	76	82	69

**Table 3:** Cost parameters

	Home isolation			Community isolation			Hospitel		
	Cost/ day	Duration of stay (day)	No. of admit	Cost/ day	Duration of stay (day)	No. of admit	Cost/ day	Duration of stay (day)	No. of admit
Mean	24.22	4.47	2,356	63.69	3.35	372	65.23	3.91	4,349
Hospitalisation Mean	541.51	9.66	96	323.95	10.00	20	622.42	7.79	162
Non- hospitalisation	21.65	4.57	2,260	60.95	6.83	352	62.45	5.00	4,187
Add-on O2 (per admit)	7.54		93	4.33		19	2.91		150

Day = patient day

**Table 4:** Cost per admission, outcome in 1000, cost per outcome, and incremental cost-effectiveness ratio

	Home isolation	Community isolation	Hospitel
Outcome: death			
Cost per admission	422	789	740
Deaths in 1000	8.49	8.06	1.61
Cost per death avoid	49.67	97.88	459.49
Incremental cost-effectiveness ratio	Ref.	866.17	46.21
Outcome: hospitalisation			
Cost per admission (before hospitalisation)	109	214	255
Hospitalisation per 1000	40.75	53.76	37.25
Cost per hospitalisation avoid	2.66	3.97	6.85
Incremental cost-effectiveness ratio	Ref.	-8.07	41.93

**Fig. 2:**
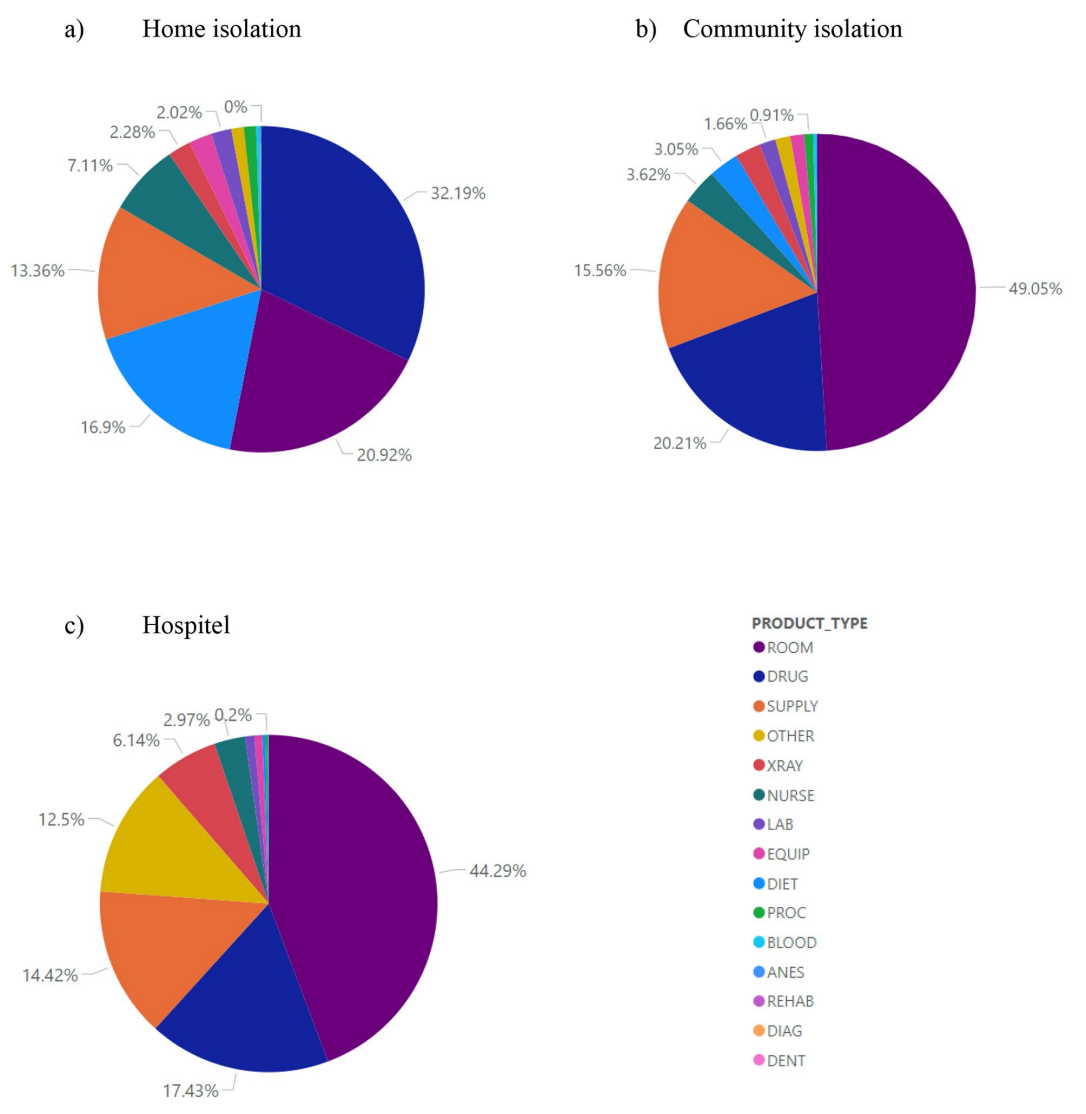
Cost by product type of each isolation strategy

Overall treatment costs were about 3.216, 0.993, and 0.294 million US$ spending for hospitel, HI, and CI patients, respectively, see Table 2. The costs per admission was highest in CI followed by hospitel and HI with the costs of 789, 740, and 422 US$, respectively. Costs per patient per day of these corresponding strategies were 76, 82, and 46 US$, in which hospitel had the highest followed by CI, then HI. Cost by product type for each isolation strategy was displayed (see Fig. 2), which suggested that costs for rooms were the major item in hospitel (44.29%) and CI (49.05%), while drug cost was the major item for HI (32.19%). Combined costs of room and drug consumed more than half for all 3 isolation strategies, i.e., 53.11, 69.26, and 61.72% for HI, CI, and hospitel, respectively.

Average durations of stay were 3.35, 3.91, and 4.47 days for CI, hospitel, and HI, respectively (see Table 3). The average costs per day for staying in these corresponding places were 63.69, 65.23 and 24.22 US$. Cost for hospitalisations highly increased for all isolation types with the average costs per day of 323.95, 622.42, and 541.51 US$, respectively, which were much higher than costs for those patients who still stayed at isolation areas with corresponding average daily costs of 60.95, 62.45, and 21.65 US$, respectively. In addition, for any patient who need oxygenation, the add-on cost was at 2.91–7.54 US$ per admission.

The terminal node (blue triangle) of all 3 decision trees presented the total cost of each arm with the final outcome as death or home (alive). When rolling-back the tree, the cost would be calculated back, weighted with its probability at each choice node (green circle). Cost per admission was highest in CI, followed by hospitel, and HI with average cost/admission of 789, 740, and 422 US$, respectively, see Table 4. Incremental cost-effectiveness ratios (ICER) were further estimated for hospitalisation and death using HI as the reference. For hospitalisation, the ICERs were 41.93 and − 8.07 US$ for hospitel and CI, respectively to avoid one hospitalisation in 1,000 patients when compared to HI, indicating CI was not cost-effective since more cost was spent for worse outcome i.e., higher rate of hospitalisation. Whereas the hospitel was cost-effective relative to HI if the willingness to pay is more than 41.93 US$ per one case avoided. For death outcome, both CI and hospitel strategies could save more lives, although the cost for one case avoided from CI strategy (866.17 US$) was about 20 times more expensive than hospitel (46.21 US$) when compared to HI.

## Discussion

This study was conducted to investigate the cost-effectiveness of HI, CI and hospitel isolations using a decision tree model. This study found that cost per each isolation was lower in HI relative to hospitel and CI, in which CI was the most expensive measure. In addition, CI was not cost-effective with worse outcomes compared to the other two strategies. Cost per hospitalisation and death avoidance were lowest in HI. Hospitel had lower hospitalisation and death rates, although required more expense compared to HI and CI. Thus, cost-benefit analysis or cost-utility analysis for these two strategies might be useful in the future.

For clinical outcomes, although hospitalisation rates among hospitel, HI and CI were not clinically significant (3.72%, 4.07% and 5.38%, respectively), death rate of HI and CI were approximately 5 times higher than hospitel. This might be due to in HI patients had higher proportion of underlying diseases, which also affected disease progression, than hospitel patients. Although similar treatment protocol was provided for all isolation strategies, the adherence to protocol of CI patients might be different relative to HI and hospitel; this might be due to the CI was operated by local governance under supervision of hospital staffs, whereas patients under HI and hospitel were directly followed up by staffs of Ramathibodi Hospital. Therefore, difference of care provided to patient might have occurred in the real world situation. Consequently, these lead to the higher death rate which was found in CI.

For economic aspect, this study’s findings were in line with previous study from Australia, in which HI was shown to be more cost-effective, compared to hospitel [27]. The HI was designed as housing facilities for people with confirmed COVID-19, who did not require hospital care, which could reduce in-patient room cost and nursing care cost. HI strategy is demonstrated by its ability to provide good psychological and social support with minimal requirement of logistic support. The majority of patients under HI recovered successfully and only a minority of them (4.07%) required hospitalisation. For CI, most of the expenses were used for reconstructing public building to be temporary shelter. Additionally, fewer patients were suitable for stay in CI. These might lead to higher cost per number of admissions. Moreover, higher deaths in CI might be due to adherence of taking care by local government personnel under hospital protocol which was different from the other 2 strategies, as aforementioned. These lead to higher hospitalisation and deaths. Finally, ICER indicated CI was not cost effective, compared to hospitel and HI.

Based on this study’s results, both HI and CI strategies could help to deal with a surge of COVID-19 infected cases in low and middle income countries like Thailand, where there are limitations of healthcare infrastructure and funding. However, several issues should be addressed. First, categorising severity should be standardised and auditing should be implemented to prevent inappropriate selection of patients to isolation strategies. Second, the concept of infectious controls (e.g., isolation area, personal protective equipment, disinfection process, waste management, etcetera) should be emphasised and implemented to all kinds of isolations to prevent secondary transmission of disease to household and community members. Third, standard training and testing programs about disease, care process and infectious control should be provided for local government healthcare officers or volunteers or care givers. Finally, intermittent audit should be implemented to ensure that appropriate medical care is provided for patients in CI.

As for our knowledge, this study is the first study to compare cost-effectiveness between HI, CI and hospitel isolation based on the real world situation in middle income country. However, some limitations were also found. First, this study did not include and consider secondary transmission as the outcomes of interest, because the database contained only patients who were confirmed as Covid-19 infections, not other family members who were also infected but might attend other hospitals. This study’s data was from a Southeast Asian country which still lacks the evidence of cost-effective analysis in COVID-19 measures. Second, this cost-effective analysis was performed based on hospital perspective, not societal perspective; thus, did not account for other relevant unknown costs such as community preparation, potential side effects and indirect-cost from quarantine, etcetera. The hospital perspective in this study would be helpful for health policy makers to make decisions for investment about quarantine policy during COVID-19 outbreak and prepare for responses to other new emerging diseases.

## Conclusion

This cost-effectiveness analysis provided the insight of isolation strategies for patients with COVID-19 based on hospital perspective. Cost per isolation is lowest in HI followed by hospitel and CI. The results showed that HI may be a cost-effective isolation measure, both in terms of preventing hospitalisation and death, in developing countries with limited resources. However, some issued should be emphasised including patient monitoring process, adherence to treatment protocol, and implementing infectious controls to prevent secondary household transmission.

### Electronic supplementary material

Below is the link to the electronic supplementary material.

Supplementary Material 1

Supplementary Material 2

## Data Availability

The authors confirm that the data supporting the findings of this study are available within the article and its supplementary materials.

## References

[1] Wu F, Zhao S, Yu B (2020). A new coronavirus associated with human Respiratory Disease in China. Nature.

[2] Li Q, Guan X, Wu P (2020). Early Transmission Dynamics in Wuhan, China, of Novel Coronavirus-Infected Pneumonia. N Engl J Med.

[3] Talukder A, Razu SR, Alif SM (2022). Association between symptoms and severity of Disease in Hospitalised Novel Coronavirus (COVID-19) patients: a systematic review and Meta-analysis. J Multidiscip Healthc.

[4] Zhang JJ, Dong X, Cao YY (2020). Clinical characteristics of 140 patients infected with SARS-CoV-2 in Wuhan. China Allergy.

[5] Hu Y, Sun J, Dai Z (2020). Prevalence and severity of corona virus Disease 2019 (COVID-19): a systematic review and meta-analysis. J Clin Virol.

[6] Ma Q, Liu J, Liu Q (2021). Global percentage of asymptomatic SARS-CoV-2 Infections among the Tested Population and individuals with confirmed COVID-19 diagnosis: a systematic review and Meta-analysis. JAMA Netw Open.

[7] Carlos WG, Dela Cruz CS, Cao B (2020). Novel Wuhan (2019-nCoV) coronavirus. Am J Respir Crit Care Med.

[8] Cai J, Sun W, Huang J (2020). Indirect Virus transmission in cluster of COVID-19 cases, Wenzhou, China, 2020. Emerg Infect Dis.

[9] Han Y, Yang H (2020). The transmission and diagnosis of 2019 novel coronavirus Infection Disease (COVID-19): a Chinese perspective. J Med Virol.

[10] Epidemiology Working Group for Ncip Epidemic Response CCfDC (2020). Prevention. [The epidemiological characteristics of an outbreak of 2019 novel coronavirus Diseases (COVID-19) in China]. Zhonghua Liu Xing Bing Xue Za Zhi.

[11] Zhang XM, Zhou HE, Zhang WW (2020). Assessment of Coronavirus Disease 2019 Community Containment Strategies in Shenzhen, China. JAMA Netw Open.

[12] World Health O (2020). Clinical management of COVID-19: interim guidance, 27 May 2020.

[13] Fuchs JD, Carter HC, Evans J (2021). Assessment of a hotel-based COVID-19 isolation and Quarantine Strategy for persons experiencing homelessness. JAMA Netw Open.

[14] MOPH enphasizes no. outbreak of Novel Corona virus in Thailand, ensuring Thailand’s preventive measures for emerging disease, early detection, early treatment. In: Emergency Operation Center DoDC, ed. Press Release 2020.

[15] Human transmissin of coronavirus confirmed in Thailand Bangkok Post 2020.

[16] Declaration of an Emergency Situation in all areas of the Kingdom of Thailand, 2020.

[17] Wancharoen S. City Hall to open hospitels with over 4,400 beds. Bangkok Post. 2021.

[18] Wipatayotin A. Ministry okays home isolation plan: aims to free up beds for more seriously ill Bangkok Post. 2021.

[19] Thailand unveils plans. To cope with Omicron COVID variant outbreak. Thai PBS World; 2021.

[20] Bangprapa M. ‘Community isolation’ threat Bangkok Post. 2021.

[21] Vandepitte S, Alleman T, Nopens I (2021). Cost-effectiveness of COVID-19 Policy measures: a systematic review. Value Health.

[22] Sheinson D, Dang J, Shah A (2021). A cost-effectiveness Framework for COVID-19 treatments for hospitalized patients in the United States. Adv Ther.

[23] Cleary SM, Wilkinson T, Tamandjou Tchuem CR (2021). Cost-effectiveness of intensive care for hospitalized COVID-19 patients: experience from South Africa. BMC Health Serv Res.

[24] Gandjour A (2021). How many intensive care beds are justifiable for hospital pandemic preparedness? A cost-effectiveness analysis for COVID-19 in Germany. Appl Health Econ Health Policy.

[25] Melia A, Lee D, Mahmoudi N et al. Cost-effectiveness analysis of COVID-19 Case Quarantine Strategies in Two Australian States: New South Wales and Western Australia. Journal of Risk and Financial Management. 2021.

[26] Bank of Thailand., Daily exchange rate, https://www.bot.or.th/english/_layouts/application/exchangerate/exchangerate.aspx. (Accessed on September 16, 2022).

[27] Royal Thai Government Gazette. In: Health MoP, editor, 2019, 30 August.

