# Supplementary material for: Cost-effectiveness analysis of isolation strategies for asymptomatic and mild symptom COVID-19 patients
Source: Cost Eff Resour Alloc. 2023 Nov 9;21:85. doi: 10.1186/s12962-023-00497-x (PMC10636943; doi:10.1186/s12962-023-00497-x)
Supplement: Supplementary file 1 — Supplementary Material 1 [file 12962_2023_497_MOESM1_ESM.docx]

Supplement Figure 1. A causal conceptual frame work of Covid-19 isolations and disease progression.
