# Supplementary material for: Cost-effectiveness analysis of isolation strategies for asymptomatic and mild symptom COVID-19 patients
Source: Cost Eff Resour Alloc. 2023 Nov 9;21:85. doi: 10.1186/s12962-023-00497-x (PMC10636943; doi:10.1186/s12962-023-00497-x)

Supplement Figure 2. Three isolation strategies cost-effectiveness analysis from decision tree model

a) Home isolation


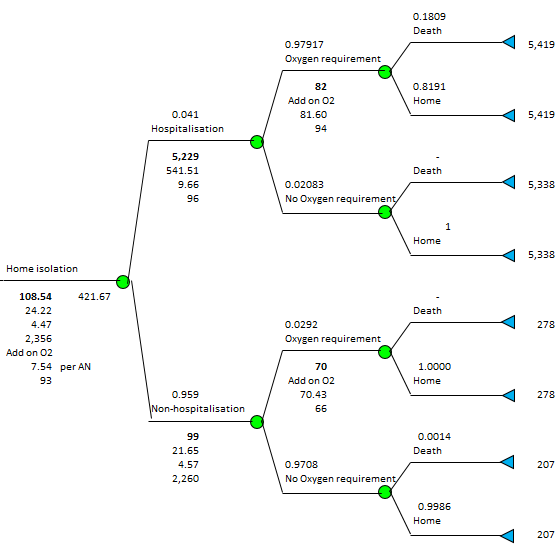


Non-hospitalisation

b) Community isolation


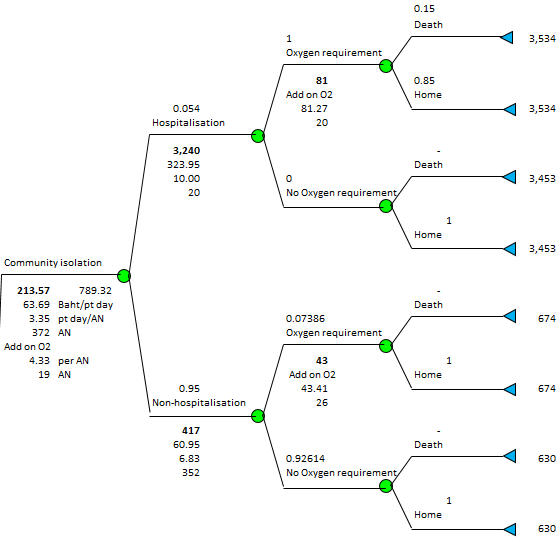


c) Hospitel


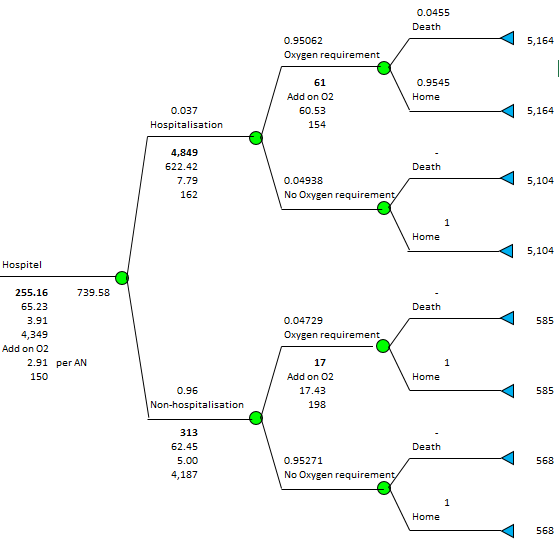

Supplement: Supplementary file 2 — Supplementary Material 2 [file 12962_2023_497_MOESM2_ESM.docx]
